# Astragalin Inhibits Cigarette Smoke-Induced Pulmonary Thrombosis and Alveolar Inflammation and Disrupts PAR Activation and Oxidative Stress-Responsive MAPK-Signaling

**DOI:** 10.3390/ijms22073692

**Published:** 2021-04-01

**Authors:** Yun-Ho Kim, Min-Kyung Kang, Eun-Jung Lee, Dong Yeon Kim, Hyeongjoo Oh, Soo-Il Kim, Su Yeon Oh, Woojin Na, Jae-Hoon Shim, Il-Jun Kang, Young-Hee Kang

**Affiliations:** Department of Food and Nutrition and Korean Institute of Nutrition, Hallym University, Chuncheon 24252, Korea; royalskim@hallym.ac.kr (Y.-H.K.); mitholy@hallym.ac.kr (M.-K.K.); reydmswjd@naver.com (E.-J.L.); ehddus3290@naver.com (D.Y.K.); ohhyeongju@gmail.com (H.O.); ky4850@naver.com (S.-I.K.); suy0411@naver.com (S.Y.O.); nsm0729@hanmail.net (W.N.); jhshim@hallym.ac.kr (J.-H.S.); ijkang@hallym.ac.kr (I.-J.K.)

**Keywords:** astragalin, thrombin, cigarette smoking, pulmonary thrombosis, alveolar inflammation

## Abstract

Epidemiological evidence shows that smoking causes a thrombophilic milieu that may play a role in the pathophysiology of chronic obstructive pulmonary disease (COPD) as well as pulmonary thromboembolism. The increased nicotine level induces a prothrombotic status and abnormal blood coagulation in smokers. Since several anticoagulants increase bleeding risk, alternative therapies need to be identified to protect against thrombosis without affecting hemostasis. Astragalin is a flavonoid present in persimmon leaves and green tea seeds and exhibits diverse activities of antioxidant and anti-inflammation. The current study investigated that astragalin attenuated smoking-induced pulmonary thrombosis and alveolar inflammation. In addition, it was explored that molecular links between thrombosis and inflammation entailed protease-activated receptor (PAR) activation and oxidative stress-responsive mitogen-activated protein kinase (MAPK)-signaling. BALB/c mice were orally administrated with 10–20 mg/kg astragalin and exposed to cigarette smoke for 8 weeks. For the in vitro study, 10 U/mL thrombin was added to alveolar epithelial A549 cells in the presence of 1–20 µM astragalin. The cigarette smoking-induced the expression of PAR-1 and PAR-2 in lung tissues, which was attenuated by the administration of ≥10 mg/kg astragalin. The oral supplementation of ≥10 mg/kg astragalin to cigarette smoke-challenged mice attenuated the protein induction of urokinase plasminogen activator, plasminogen activator inhibitor-1and tissue factor, and instead enhanced the induction of tissue plasminogen activator in lung tissues. The astragalin treatment alleviated cigarette smoke-induced lung emphysema and pulmonary thrombosis. Astragalin caused lymphocytosis and neutrophilia in bronchoalveolar lavage fluid due to cigarette smoke but curtailed infiltration of neutrophils and macrophages in airways. Furthermore, this compound retarded thrombin-induced activation of PAR proteins and expression of inflammatory mediators in alveolar cells. Treating astragalin interrupted PAR proteins-activated reactive oxygen species production and MAPK signaling leading to alveolar inflammation. Accordingly, astragalin may interrupt the smoking-induced oxidative stress–MAPK signaling–inflammation axis via disconnection between alveolar PAR activation and pulmonary thromboembolism.

## 1. Introduction

Epidemiological evidence shows that smoking is related to chronic obstructive pulmonary disease (COPD) with chronic bronchitis and emphysema [1]. It is known that smoking develops blood clots/thrombi and subsequent pulmonary thromboembolism [2,3]. Pulmonary thrombosis takes place when blood clots become lodged in pulmonary arteries [4,5]. Indeed, smoking causes a thrombophilic milieu that may play a role in the pathophysiology of COPD as well as pulmonary thromboembolism [2,6]. Pulmonary thromboembolism is potentially deadly in patients with COPD and is highly frequent during COPD exacerbations [7,8]. Risk factors for pulmonary thromboembolism include prolonged immobility, medical conditions, such as cancer and heart diseases, being overweight, supplemental estrogen, pregnancy and COVID-19 [9,10,11]. It has been reported that smoking stimulates the incidence of coagulation abnormalities and elevated plasma fibrinogen levels [2]. However, it is obscure that abnormal blood coagulation contributes directly to COPD’s pathophysiology [2]. Nevertheless, the increased levels of nicotine due to smoking induce a prothrombotic status in smokers through enhancing platelet-dependent thrombogenesis [12]. Exposure to nicotine enhances coagulation and induces plasminogen activator inhibitor-1 (PAI-1), a major regulator of fibrinolysis [2].

It has been revealed that excessive activation of coagulation occurs in the setting of inflammation [13]. The potential influence of inflammation on coagulation contributes to thrombogenesis in all smokers [2]. The molecular and cellular pathways in links between coagulation and inflammation have been reported [13]. The coagulation protease thrombin displays hemostasis and thrombosis and profound inflammatory responses through activation of the coagulation–inflammation axis [14]. Protease-activated receptors (PAR) are expressed on platelets, leukocytes and endothelial cells and modulate the responses to coagulation proteases, such as thrombin during hemostasis, thrombosis and inflammatory states [15]. The PAR activation is involved in the development of chronic inflammatory diseases [16]. Cigarette smoking impairs PAR-1-mediated fibrinolytic function, indicating increased thrombotic risk in smokers [17]. PAR-2 is highly expressed and causes inflammation in the airways of smokers [18]. Thrombin-mediated PAR activation stimulates diverse functions, such as granule releases, morphological change and aggregation of platelets, mobilization of intracellular calcium, and translocation of adhesion molecules [15]. Thrombi results in tissue ischemia and fibrin degradation, thus enhancing inflammation of activated platelets [16]. Accordingly, targeting against thrombin or PAR may diminish inflammatory diseases. In addition, thrombin and platelets may be prime targets for anticoagulant therapies against thrombosis.

Based on a large body of clinical trials, several novel drugs reduce thrombosis through targeting thrombin or PAR-1 [19,20,21]. Since anti-coagulants increase the risk of bleeding, alternative therapies need to be identified to protect against thrombosis without affecting hemostasis. Potential anti-inflammatory drugs would be strong candidates for targeting thrombosis connected to the inflammatory pathways triggered by thrombin and PAR-1 [15,16]. Because of the undesirable effects of antiplatelet and anticoagulant drugs, in recent years, plant compounds have been considered as alternative drugs to hamper the coagulation pathway and to reduce activation of platelets [22,23,24]. The hydroxyl groups in flavonoids are known to be crucial for optimally inhibiting platelet activities [23]. The polyphenols of cyanidin, quercetin and silybin inhibit thrombin amidolytic activity and modulate thrombin proteolytic activity [22]. It has been previously reported that the polyphenol astragalin inhibits pulmonary inflammation and airway epithelial fibrosis [25,26]. The current study examined whether naturally occurring astragalin inhibited cigarette smoking-induced pulmonary thrombosis and inflammation via blockade of PAR signaling in mice. Furthermore, the in vitro study investigated that this compound attenuated thrombin-induced PAR signaling and oxidative stress leading to alveolar inflammation in A549 cells. This study found that astragalin may be a potential agent alleviating smoking-induced oxidative stress and inflammation via disconnection between PAR activation and pulmonary thromboembolism.

## 2. Results

### 2.1. Inhibitory Effects of Astragalin on PAR Induction in Cigarette Smoke-Exposed Mice

**Figure 1:**
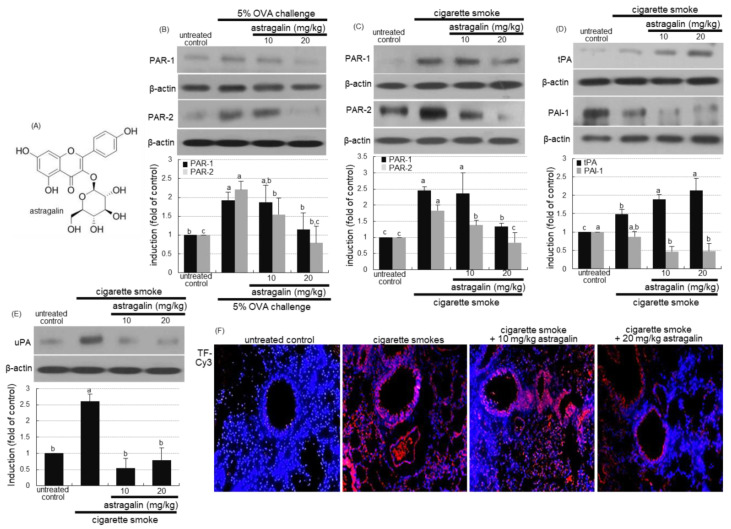
Chemical structure (**A**) and inhibition of induction of protease-activated receptor (PAR) proteins (**B**,**C**) and plasminogen activator proteins (tPA and uPA) and plasminogen activator inhibitor-1(PAI-1, (**D**,**E**)) by astragalin in lung tissues of ovalbumin (OVA)- or cigarette smoke-exposed mice. Mice were orally administrated with 10 or 20 mg/kg astragalin and exposed to cigarette smoke. Additionally, BALB/c mice were OVA-sensitized and orally supplemented with 10–20 mg/kg astragalin (**B**). Tissue extracts were subject to Western blot with a primary antibody against PAR-1, PAR-2, tPA, PAI-1 or uPA (**B**–**E**). β-Actin protein was used as an internal control. The bar graphs (mean ± SEM, *n* = 3) represent quantitative results of the upper bands obtained from a densitometer. Values in bar graphs (same achromatic colored) not sharing the same lower case alphabet letter indicate a significant difference at *p* < 0.05. The tissue factor (TF) localization was identified as Cy3-red staining in mouse lungs exposed to cigarette smoke (**F**). Nuclear staining was done with 4′,6-diamidino-2-phenylindole (blue). Each photograph is representative of four mice. Magnification: 200-fold.

This study examined whether astragalin influenced the expression of PAR-1 and PAR-2 in cigarette smoke-exposed mice. The OVA sensitization induced the expression of PAR-1 and PAR-2 in lung tissues, which was attenuated by the administration of ≥10 mg/kg astragalin (Figure 1B). In addition, the induction of PAR-1 and PAR-2 was enhanced in cigarette smoke-exposed lung tissues (Figure 1C). When 10–20 mg/kg astragalin was supplemented to cigarette smoke-inhaled mice, the induction of these proteins was diminished (Figure 1C).

### 2.2. Modulation of Plasminogen Activation by Astragalin in Cigarette Smoke-Exposed Mice

The oral supplementation of ≥10 mg/kg astragalin to cigarette smoke-exposed mice enhanced the induction of tissue plasminogen activator (tPA) in mouse lung tissues (Figure 1D). In contrast, the PAI-1 expression by cigarette smoke was diminished by treating 10–20 mg/kg astragalin to mice. It should be noted that in untreated control mice, there was weak tPA expression, but PAI-1was highly expressed (Figure 1D). Further, the cigarette smoke enhanced the lung tissue level of urokinase plasminogen activator (uPA), which was attenuated by ≥10 mg/kg astragalin (Figure 1E). Furthermore, the immunohistochemical staining revealed that astragalin reduced the tissue factor (TF) protein level highly elevated in the small airways of mice exposed to cigarette smoke (Figure 1F).

### 2.3. Blockade of Cigarette Smoke-Induced Lung Thrombosis in Mice by Astragalin

**Figure 2:**
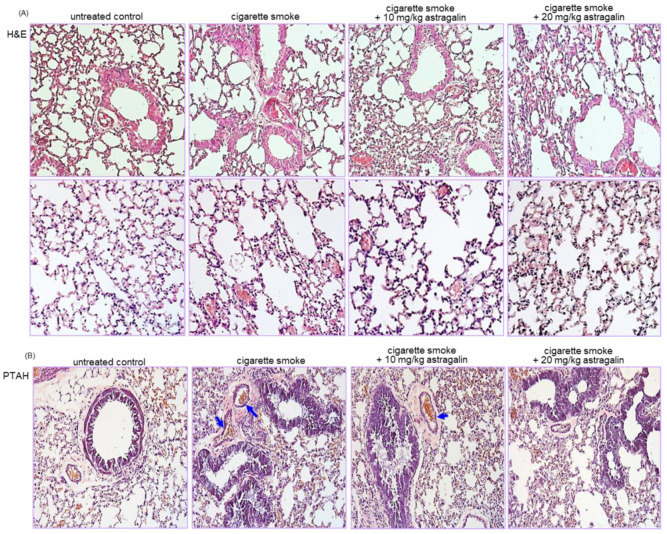
Suppressive effects of astragalin on airway injury (**A**) and thrombus formation (**B**) in airways and alveoli of cigarette smoke-exposed mice. Mice were orally administrated with 10 or 20 mg/kg astragalin and exposed to cigarette smoke. Histological examination was performed with phospho-tungstic acid hematoxylin (PTAH) and hematoxylin and eosin (H&E) staining. Tissue sections of small airways and alveoli were stained by using H&E reagent (**A**). PTAH staining showing blockade of thrombus by oral administration of astragalin in lung blood vessels of cigarette smoke-exposed mice (**B**). The blue arrows indicate thrombus formation. Each photograph is representative of four mice. Magnification: 200-fold.

This study conducted a histological examination in the airways and alveoli stained with hematoxylin and eosin (H&E). Microscopic description of the thrombi was made concerning blood cells on H&E-stained slides [27]. The lungs of the control mice showed no significant thickening of airway epithelial mucosa and rupturing of the alveolar walls (Figure 2A). In contrast, the cigarette smoke-exposed lungs exhibited airway mucosal thickening and alveolar wall rupture. When cigarette smoke-loaded mice were treated with 10–20 mg/kg astragalin, small airway thickening and alveolar emphysema were alleviated (Figure 2A).

This study further investigated that astragalin attenuated thrombus formation in pulmonary vessels of cigarette smoke-inhaled mice. A microscopic description of the thrombi was made concerning fibrin on phospho-tungstic acid hematoxylin (PTAH)-stained slides [27]. There were no thrombi in pulmonary vessels of control mice, as evidenced by PTAH staining (Figure 2B). In contrast, cigarette smoke-induced vascular thrombosis in mice (blue arrows). However, oral administration of 20 mg/kg astragalin inhibited the thrombus formation in pulmonary vessels exposed to cigarette smoke (Figure 2B). Thus, astragalin may attenuate pulmonary embolism and infarction created by cigarette smoke.

### 2.4. Inhibition of Cigarette Smoke-Induced Pulmonary Inflammation by Astragalin

**Figure 3:**
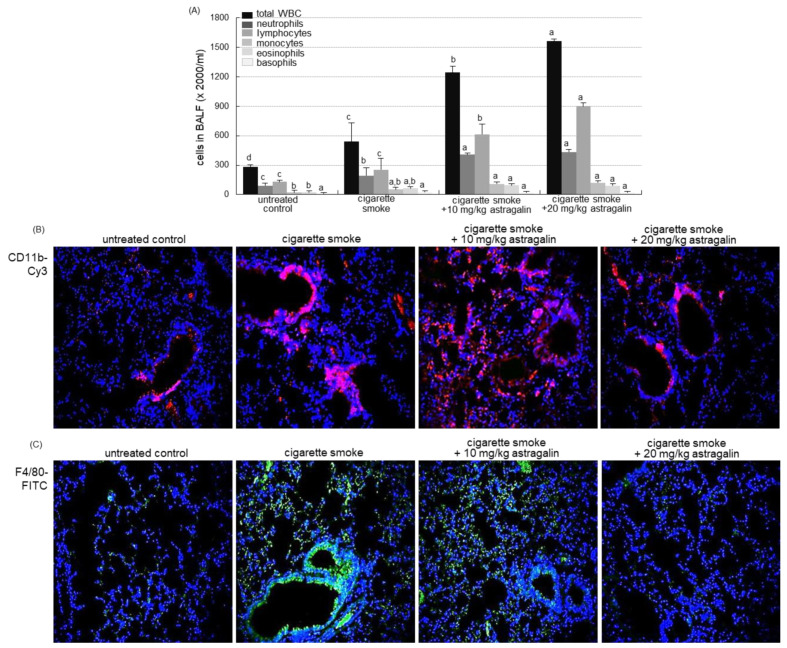
Contents of inflammatory cells in the bronchoalveolar lavage fluid (BALF, (**A**)) and infiltration of inflammatory cells (**B**) in the lungs of mice exposed to cigarette smoke. Mice were orally administrated with 10 or 20 mg/kg astragalin and exposed to cigarette smoke. Cells in BALF were counted using a Hemavet HV950 multispecies hematologic analyzer (**A**). Values in bar graphs (same achromatic colored) not sharing the same lower case alphabet letter indicate a significant difference at *p* < 0.05. For the measurement of levels of CD11b and F4/80 in mouse airways exposed to cigarette smoke, the CD11b was visualized with Cy3-red staining (**B**), and the F4/80 localization was identified as FITC-green staining (**C**). Nuclear staining was done with 4′,6-diamidino-2-phenylindole (blue). Each photograph is representative of four mice. Magnification: 200-fold.

The current study investigated that astragalin attenuated pulmonary inflammation prompted by cigarette smoke. The challenge of cigarette smoke elevated the number of total leukocytes in the bronchoalveolar lavage fluid (BALF) by ~2-fold (Figure 3A). The administration of ≥10 mg/kg astragalin further enhanced cigarette smoke-induced leukocytosis of lymphocytes, neutrophils, and monocytes (Figure 3A). These data indicate that the combination of cigarette smoke inhalation and astragalin treatment resulted in the gathering of lymphocytes and neutrophils into the BALF.

The neutrophilic marker CD11b was highly expressed around mouse airways exposed to cigarette smoke (Figure 3B). In contrast, the Cy3-red staining showed that the oral administration of 10–20 mg/kg astragalin reduced its induction in airways. In addition, the FITC-immunohistochemical staining revealed that astragalin curtailed the airway induction of the macrophage marker F4/80 by cigarette smoking (Figure 3B). Accordingly, the BALF cellularity may not reflect the status of the inflammatory process or structural derangements in lungs suffering inflammation evoked by cigarette smoke.

### 2.5. Suppressive Effects of Astragalin on Thrombin-Induced Alveolar Inflammation

**Figure 4:**
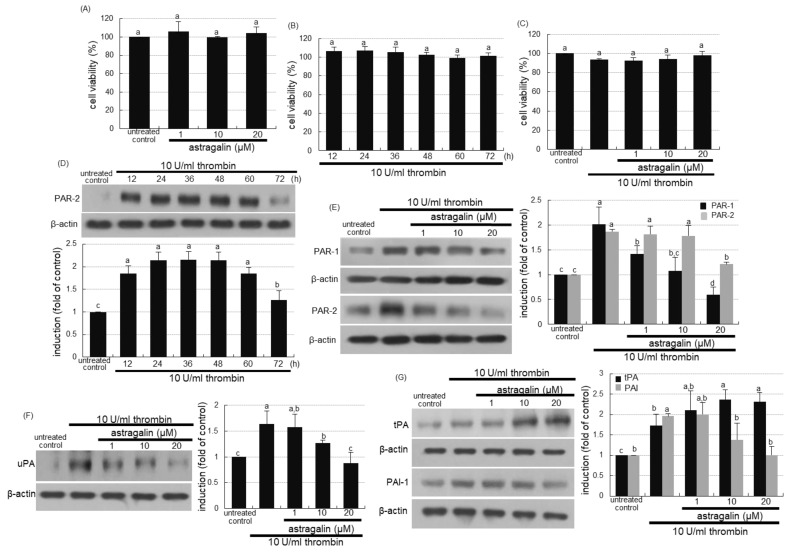
Cytotoxicity of astragalin and thrombin (**A**–**C**), and temporal expression (**D**) and inhibition (**E**) of protease-activated receptor (PAR) proteins, and inhibition of urokinase plasminogen activator (uPA, (**F**)), and modulation of tissue plasminogen activator (tPA) and plasminogen activator inhibitor-1(PAI-1, G) in thrombin-exposed A549 cells with and without 1–20 μM astragalin. Alveolar epithelial cells were incubated in media containing 10 U/mL thrombin in the absence and presence of 1–20 μM astragalin for up to 72 h. A549 cell viability (mean ± SEM, *n* = 5) was measured by using MTT assay and expressed as percent cell survival relative to untreated controls (**A**–**C**). Cell lysates were prepared for Western blot analysis with a primary antibody against PAR-1, PAR-2, uPA, tPA and PAI-1 (**D**–**G**). The bar graphs (mean ± SEM, *n* = 3) represent quantitative results of the upper left bands obtained from a densitometer. β-Actin protein was used as an internal control. Values in bar graphs (same achromatic colored) not sharing the same lower case alphabet letter indicate a significant difference at *p* < 0.05.

This study attempted to investigate that thrombi in vessels were involved in evoking alveolar inflammation, which was attenuated by treating astragalin. No alterations were observed in cell viability when either 1–20 μM astragalin or 10 U/mL thrombin was solely treated to A549 cells for 72 h (Figure 4A,B). In addition, 1–20 μM astragalin did not show cytotoxicity even in combination with 10 U/mL thrombin (Figure 4C). When 10 U/mL thrombin was loaded to A549 cells, the cellular expression of PAR-2 was highly induced up to 60 h (Figure 4D). In contrast, 20 μM astragalin diminished thrombin-induced induction of both PAR-1 and PAR-2 (Figure 4E). Cellular uPA was induced in thrombin-loaded alveolar cells, which was attenuated by submicromolar astragalin (Figure 4F). Thrombin influenced the expression of tPA and PAI-1 reciprocally (Figure 4G). The tPA expression by thrombin was further induced, but the PAI-1 expression was dose-dependently diminished by treating 1–20 μM astragalin (Figure 4G).

**Figure 5:**
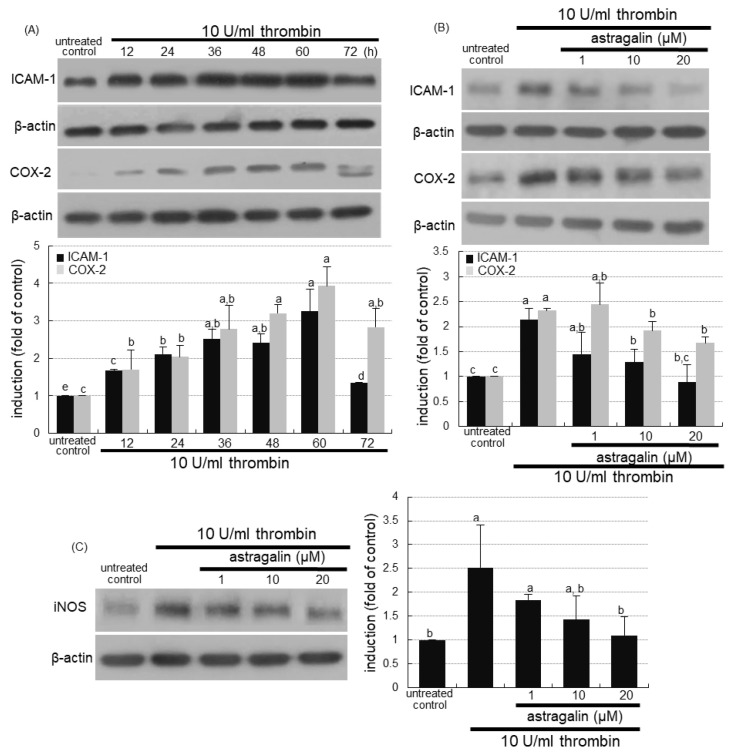
Temporal induction of alveolar inflammatory mediators by thrombin (**A**) and blockade of their induction by astragalin in thrombin-treated A549 cells (**B**,**C**). Alveolar epithelial cells were incubated in media containing 10 U/mL thrombin in the absence and presence of 1–20 μM astragalin for up to 72 h. Cell lysates were subject to Western blot analysis with a primary antibody against ICAM-1, COX-2, or iNOS. β-Actin protein was used as an internal control. The bar graphs (mean ± SEM, *n* = 3) represent quantitative results of the upper or left bands obtained from a densitometer. Values in bar graphs (same achromatic colored) not sharing the same lower case alphabet letter indicate a significant difference at *p* < 0.05.

This study examined whether thrombosis elicited alveolar inflammation, which was blocked by supplementing astragalin to alveoli. The protein levels of intracellular adhesion molecule (ICAM)-1 and cyclooxygenase (COX)-2 were highly enhanced in alveolar epithelial A549 cells for 12–60 h following the 10 U/mL thrombin loading (Figure 5A). However, astragalin inhibited thrombin-induced expression of alveolar ICAM-1 responsible for pulmonary neutrophil recruitment (Figure 5B). In addition, ≥10 μM astragalin diminished the elevated protein level of inflammatory COX-2 responsible for prostaglandin biosynthesis (Figure 5B). The induction of inducible nitric oxide synthase (iNOS) directly involved in inflammatory responses was observed in alveolar epithelial cells exposed to thrombin, and such induction was dampened by treating ≥10 μM astragalin (Figure 5C). Therefore, astragalin may inhibit pulmonary thrombosis-triggered alveolar inflammation.

### 2.6. Inhibition of MAPK Signaling in Thrombin-Exposed Alveoli by Astragalin

**Figure 6:**
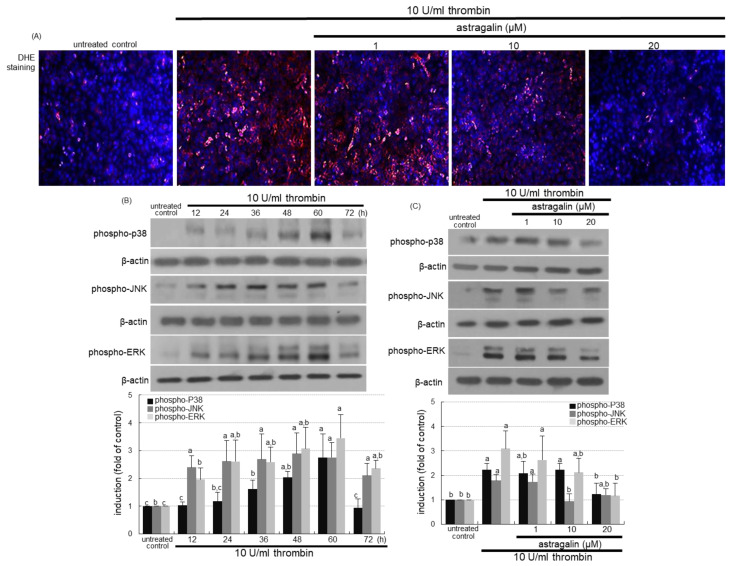
Inhibition of oxidant production (**A**), temporal activation of mitogen-activated protein kinase (MAPK) signaling (**B**) and their inhibition (**C**) by astragalin in thrombin-treated A549 cells. Alveolar epithelial cells were incubated in media containing 10 U/mL thrombin in the absence and presence of 1–20 μM astragalin for up to 72 h. For the measurement of the reactive oxygen species production, dihydroethidium (DHE) staining was conducted in thrombin-treated A549 cells (**A**). Nuclear staining was done with 4′,6-diamidino-2-phenylindole (blue). Each photograph is representative of stained cells. Magnification: 200-fold. Cell lysates were subject to Western blot analysis with a primary antibody against phospho-p38, phospho-c-Jun N-terminal kinase (JNK) or phospho-extracellular signal-regulated kinases (ERK) (**B**,**C**). β-Actin protein was used as an internal control. The bar graphs (mean ± SEM, *n* = 3) represent quantitative results of the upper bands obtained from a densitometer. Values in bar graphs (same achromatic colored) not sharing the same lower case alphabet letter indicate a significant difference at *p* < 0.05.

This study investigated that thrombosis resulted in oxidative stress leading to activation of mitogen-activated protein kinase (MAPK) signaling in alveoli. There was a strong red-fluorescent dihydroethidium (DHE) staining in 10 U/mL thrombin-stimulated alveolar cells (Figure 6A). However, the treatment of 1–20 μM astragalin markedly attenuated the reactive oxygen species (ROS) production in thrombin-loaded A549 cells. As expected, the MAPK signaling of p38, c-Jun N-terminal kinase (JNK) and extracellular signal-regulated kinases (ERK) was activated for up to 60 h following the thrombin challenge to A549 cells (Figure 6B). Accordingly, pulmonary thrombosis led to oxidative stress and MAPK activation responsible for pulmonary inflammation. However, 20 μM astragalin inhibited their phosphorylation in alveolar epithelial cells (Figure 6C).

**Figure 7:**
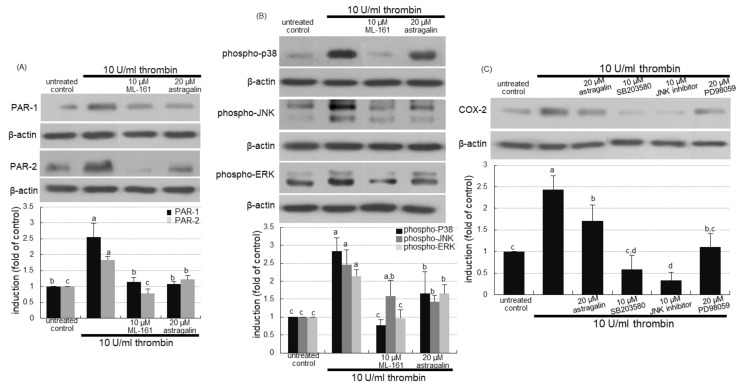
Inhibition of activation mitogen-activated protein kinase (MAPK) by protease-activated receptor (PAR) inhibitor (**A**,**B**), and cyclooxygenase (COX)-2 inhibition by MAPK inhibitors (**C**) in thrombin-treated A549 cells. Alveolar epithelial cells were incubated in media containing 10 U/mL thrombin in the presence of 10 μM ML-161, 20 μM astragalin, or 10–20 μM MAPK inhibitors for up to 72 h. Cell lysates were subject to Western blot analysis with a primary antibody against PAR-1, PAR-2, phospho-p38, phospho-c-Jun N-terminal kinase (JNK), phospho-extracellular signal-regulated kinases (ERK) or COX-2. β-Actin protein was used as an internal control. The bar graphs (mean ± SEM, *n* = 3) represent quantitative results of the upper bands obtained from a densitometer. Values in bar graphs (same achromatic colored) not sharing the same lower case alphabet letter indicate a significant difference at *p* < 0.05.

This study further examined whether the PAR-1 inhibition interrupted activation of MAPK signaling and subsequent inflammation in alveoli. As expected, the PAR-1 inhibitor ML-161 abolished the induction of PAR-2 as well as PAR-1 (Figure 7A). The PAR inhibition highly attenuated alveolar MAPK activation of P38, JNK and ERK inflamed by 10 U/mL thrombin (Figure 7B). Further, this study investigated that the blockade of MAPK signaling inhibited alveolar inflammation evoked by thrombin. The COX-2 induction in the presence of thrombin was highly attenuated by respective MAPK inhibitors of p38, JNK and ERK (Figure 7C). Accordingly, alveolar inflammation may entail MAPK signaling triggered by cigarette smoke-associated thrombosis.

## 3. Discussion

**Figure 8:**
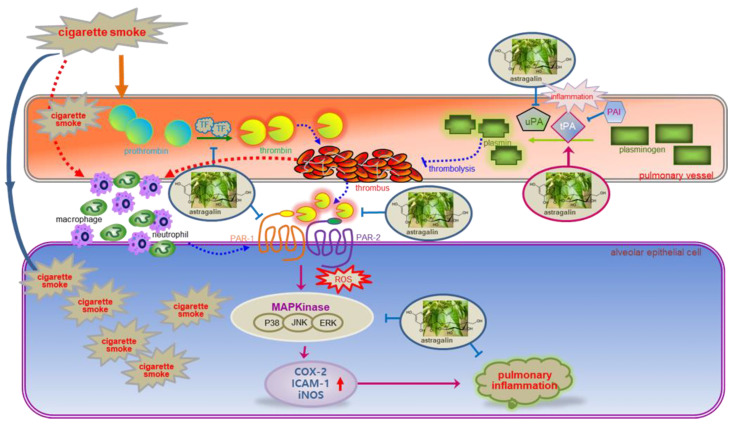
Schematic diagram showing the inhibitory effects of astragalin on astragalin may inhibit pulmonary thrombus-derived inflammation involving PAR-triggered oxidative stress-MAPK signaling. As depicted, astragalin attenuated thrombus formation and pulmonary inflammation. The symbol ⱶ indicates sites of inhibition manifested by astragalin, and the symbol → indicates activation.

Eight major findings were extracted from this study. (1) The OVA sensitization and cigarette smoking-induced the expression of PAR-1 and PAR-2 in lung tissues, which was attenuated by the administration of ≥10 mg/kg astragalin. (2) The oral supplementation of ≥10 mg/kg astragalin to cigarette smoke-exposed mice attenuated the protein induction of uPA, PAI-1and TF and instead enhanced the induction of tPA in lung tissues. (3) The astragalin treatment alleviated cigarette smoke-induced lung emphysema and pulmonary thrombosis. (4) Astragalin caused lymphocytosis and neutrophilia in BALF due to cigarette smoke but curtailed infiltration of neutrophils and macrophages in airways. (5) Submicromolar astragalin diminished the thrombin induction of PAR proteins in alveolar cells. (6) Astragalin dampened alveolar inflammation by inhibiting the expression of ICAM-1, COX-2 and iNOS induced by thrombin. (7) Treating astragalin attenuated the ROS production in thrombin-exposed alveolar cells. (8) Astragalin interrupted PAR proteins-activated MAPK signaling leading to alveolar inflammation. Accordingly, astragalin may inhibit smoking-induced oxidative stress and block the MAPK signaling–inflammation axis via disconnection between alveolar PAR activation and pulmonary thromboembolism (Figure 8).

The detrimental effects of cigarette smoking concerning cardiovascular diseases are well established [28,29]. However, the association between cigarette smoking and venous thromboembolism remains unclear. Based on the epidemiological evidence, smoking stimulates platelet-dependent coagulation and thrombogenesis, resulting in blood clots and subsequent pulmonary embolism [2,3,12]. In addition, smoking causes chronic bronchitis and emphysema, characteristic of COPD [1]. Pulmonary thromboembolism entails lodging of blood clots/thrombi in pulmonary arteries formed inside deep veins in the legs [7,8]. Thrombin, known as an active plasma coagulation factor II, plays a crucial role in the hemostatic process and regulates blood coagulant activity [30]. Smoking engenders a thrombophilic milieu, which may play a role in the pathophysiology of COPD [2,6]. This study showed that cigarette smoke airway thickening, airway wall rupture and alveolar emphysema along with pulmonary thrombosis. One study shows that the nicotine load enhances coagulation by inducing PAI-1 [2]. Increased PAI-1 expression promotes alveolar epithelial apoptosis and exacerbates lung inflammation following exposure to passive cigarette smoke [31]. In addition, tPA may attenuate ventilator-induced lung injury in a rat model through counteracting PAI [32]. Interestingly, this study showed that the tPA expression was enhanced by cigarette smoke with a reciprocal reduction of the PAI-1 expression. One can assume that cigarette smoke may induce tPA to alleviate pulmonary thromboembolism. On the other hand, tobacco smoke induces the uPA expression leading to the increase in plasmin-dependent degradation of matrix proteins and cell migration [33]. In this study, cigarette smoke-induced the expression of uPA in mouse lung tissues. The induction of uPA caused by cigarette smoke may contribute to airway wall rupture and alveolar emphysema. Accordingly, cigarette smoke may arouse thrombophilic embolism and ensure uPA-dependent alveolar emphysema.

Molecular crosstalk exists between inflammation and coagulation in a reciprocal manner, whereby inflammation leads to activation of coagulation, and coagulation considerably affects inflammatory activity [13,34]. It has been reported that TF is the main initiator of the extrinsic coagulation pathway and plays a central role via producing proinflammatory cytokines, thereby initiating coagulation and downstream cellular signaling pathways [34,35]. This study showed that cigarette smoke enhanced the lung tissue level of TF. Accordingly, the reciprocal aggravation of inflammation and TF-initiated coagulation by cigarette smoke may contribute to thrombogenesis. The TF expression entails the generation of thrombin following coagulation activation [34]. Thrombin may affect specific receptors of inflammation-responsive cells and thereby modulate the inflammatory response [34]. Indeed, thrombin caused alveolar inflammation along with the expression of neutrophilic ICAM-1 and proinflammatory COX-2 via the induction of alveolar PAR proteins. It was speculated that PAR proteins provide the molecular link between coagulation and inflammation [13]. In addition, thrombin produced ROS in alveolar cells via activation of MAPK signaling. Accordingly, cigarette smoking may cause pulmonary coagulation-associated thrombophilic inflammation involving oxidative stress-triggered MAPK signaling. Targeting against thrombin and/or PAR and unraveling the molecular link between coagulation and inflammation may help developing new strategies to ameliorate the detrimental thrombosis and pulmonary thromboembolism due to smoking [35].

Anticoagulation is the mainstay of pulmonary thromboembolism and deep venous thrombosis, which are the most important manifestations of venous thromboembolism [36]. Several studies have shown that anticoagulants reduce thrombosis through targeting against thrombin or PAR proteins [16,19,37]. Anticoagulants, such as vitamin K-antagonists and direct-acting oral anticoagulants, have adverse side effects, including bleeding, vascular calcification, and non-hemostatic vascular effects [37]. Thus, the increase in cardiovascular incidents requires the introduction of natural anticoagulants that would be effective in treating and preventing thrombosis without affecting hemostasis. Various substances of natural origin influence the mechanisms of thrombosis in diverse ways, affecting the coagulation system and platelet activation and aggregation [38]. Astragalin inhibited cigarette smoke-induced PAR signaling in lung tissues, indicating that this compound may deter thrombin activity and ensuing pulmonary thromboembolism. One study shows that phenolic compounds of cyanidin, quercetin and silybin hamper amidolytic activity of thrombin and modulate its proteolytic activity [22]. Additionally, astragalin diminished cigarette smoke-induced and uPA-mediated lung tissue damage. As expected, astragalin suppressed thrombin-stimulated alveolar induction of PAR proteins and enhanced tPA induction along with concurrent reduction of PAI-1. Since oxidative stress promotes thrombosis [39], natural compounds that reduce the level of oxidative stress may be candidates for treating thrombotic complications [38,40]. This study found that astragalin inhibited thrombin-induced ROS production and ensuing MAPK signaling, which would be a safe and effective novel antithrombotic pharmacotherapy.

A growing literature has shown that pulmonary thromboembolism is associated with systemic inflammation and hyper-coagulability [41]. Since inflammation and thrombosis are interrelated [42], the upregulation of inflammatory mediators occurs during acute thrombosis [13,14]. Accordingly, the potential value of anti-inflammatory compounds may contribute to the treatment of thrombosis. This study found that astragalin attenuated thrombin-induced alveolar inflammation involving COX-2 induction by disturbing the production of inflammatory mediators of neutrophils and macrophages. The current finding suggests that astragalin may sever a PAR-dependent connection between TF-initiated pulmonary thrombosis and oxidative stress-elicited alveolar inflammation in cigarette smoke-induced thrombotic milieu. Flavonoids abrogate hyperactivation of platelets and atherothrombosis by inhibiting excessive TF availability in the endothelium [24,38]. The plant flavonoid fisetin blunts oxidative stress, inflammation, and tissue damage in rat lungs induced by cigarette smoke [43]. Our previous studies revealed that astragalin inhibited pulmonary inflammation and airway epithelial fibrosis [25,26]. Collectively, astragalin is a multifaceted compound with diverse pharmacological applications, such as anti-inflammatory and antioxidant properties [44].

## 4. Materials and Methods

### 4.1. Chemicals

RPMI and thrombin were obtained from Sigma-Aldrich Chemical (St. Louis, MO, USA), as were all other reagents unless specifically stated elsewhere. Fetal bovine serum (FBS), penicillin–streptomycin and trypsin-EDTA were purchased from the Lonza (Walkersville, MD, USA). ML-161was provided from Sigma-Aldrich chemical. Rabbit polyclonal antibodies of ICAM-1, TF, tPA, uPA, goat polyclonal COX-2 antibody, and mouse monoclonal iNOS antibody were purchased from the Santa Cruz Biotechnology (Dallas, TX, USA). Rabbit polyclonal antibodies of phospho-p38, phospho-ERK and phospho-JNK were obtained from Cell Signaling Technology (Beverly, MA, USA). Rabbit polyclonal antibodies of PAR-1, PAR-2 and PAI-1, and rat polyclonal F4/80 were provided by Abcam (Cambridge, UK). Horseradish peroxidase (HRP)-conjugated goat anti-rabbit IgG, chicken anti-rat IgG and goat anti-mouse IgG were purchased from Jackson Immuno-Research Laboratories (West Grove, PA, USA). Mouse monoclonal β-actin antibody was obtained from Sigma-Aldrich Chemicals. Essential fatty acid-free bovine serum albumin (BSA) and skim milk were supplied by the Becton Dickinson Company (Sparks, MD, USA). 4′,6-Diamidino-2-phenylindole (DAPI) was obtained from Santa Cruz Biotechnology.

Astragalin was dissolved in dimethyl sulfoxide (DMSO, 25 mM stock solution) for live culture with cells; a final culture concentration of DMSO was ≤0.5%.

### 4.2. Animal Experiments

Six-week-old male BALB/c mice (Hallym University Breeding Center for Laboratory Animals) were used in this study. Mice were kept on a 12 h light/12 h dark cycle at 23 ± 1 °C with 50 ± 10% relative humidity under specific-pathogen-free circumstances, fed a non-purified diet, and provided with water ad libitum at the animal facility of Hallym University. The present study was approved by the Hallym University Institutional Review Board and Committee on Animal Experimentation (Hallym 2019–50) and conducted in compliance with the University’s guidelines for the care and use of laboratory animals.

Mice were acclimatized for 1 week before beginning the experiments. Mice were divided into five subgroups (*n* = 9–10 for each subgroup). Astragalin solution (containing 10–20 mg/kg BW) was orally administrated to mice via oral gavage once a day (5 days/week) for 8 weeks. In an hour, mice were exposed to the smoke of research cigarettes (11 mg tar and 0.7 mg nicotine/cigarette) for 30 min in a specially designed chamber once a day for 8 weeks. Research cigarettes (3R4F, 11 mg tar and 0.7 mg nicotine per cigarette) were obtained from the University of Kentucky (Lexington, KY, USA). All the mice were killed with an anesthetic (0.3 g/kg avertin and 8 μL/kg *tert*-amyl alcohol). The trachea was cannulated, and both lungs and airways were rinsed in 1 mL PBS to collect BALF. The numbers of inflammatory cells were determined using a Hemavet HV950 multispecies hematologic analyzer (Drew Scientific, Oxford, CT, USA). The right lungs were collected, frozen in liquid nitrogen, and kept at −80 °C until used for Western blotting. Left lungs were preserved and fixed in 4% paraformaldehyde and then used for immunohistochemical analyses.

### 4.3. Western Blot Analysis

Mouse lung tissue extracts and A549 cell lysates were prepared in 1 mM Tris-HCl (pH 6.8) lysis buffer containing 10% sodium dodecyl sulfate (SDS), 1% glycerophosphate, 0.1 mM Na_3_VO_4_, 0.5 mM NaF and protease inhibitor cocktail. Tissue extracts and cell lysates containing equal amounts of proteins were electrophoresed on 8–15% SDS–PAGE and transferred onto a nitrocellulose membrane. Blocking a nonspecific binding was performed using either 3% fatty acid-free BSA or 5% nonfat dry skim milk for 3 h. The membrane was incubated overnight at 4 °C with a specific primary antibody of PAR-1, PAR-2, tPA, uPA, PAI-1, ICAM-1, COX-2, iNOS, phospho-p38, phospho-ERK, or phospho-JNK. The membrane was then applied to a secondary antibody conjugated to HRP for 1 h. Following triple washing, the target proteins were determined using the Immobilon Western Chemiluminescent HRP substrate (Millipore Corp., Billerica, MA, USA) and the Agfa medical X-ray film blue (Agfa HealthCare NV, Mortsel, Belgium). Incubation with β-actin antibody was conducted for the comparative control.

### 4.4. Immunohistochemical Staining

Tissue samples from the lung were collected and fixed in 10% neutral buffered formalin, which was further processed in an automated tissue processor and embedded in paraffin wax before triplicate sections were prepared. Paraffin-embedded tissue sections (5 μm thickness) of small airways and alveoli were deparaffinized and hydrated to conduct immunofluorescent histochemical analyses. The sections were preincubated in a boiling sodium citrate buffer (10 mM sodium citrate, 0.05% Tween-20, pH 6.0) for antigen retrieval. The tissues were blocked with 5% BSA in phosphate-buffered saline for 1 h. A specific primary antibody against TF, CD11b or F4/80, was incubated overnight with the sectioned tissues. Subsequently, the tissue sections were incubated for 1 h with Cy3-conjugated anti-rabbit IgG or FITC-conjugated anti-rat IgG. For identification of nuclei, the fluorescent nucleic acid dye of DAPI was applied for 10 min. Stained tissues were mounted on slides using a mounting medium (Vector Laboratories, Burlingame, CA, USA). Images of each slide were obtained with an optical microscope Axioimager system equipped for fluorescence illumination (Zeiss, Gottingen, Germany).

### 4.5. Staining with H&E

For the histopathological analyses of airways, small airways and alveolar specimens obtained at the end of the experiments were fixed in 10% neutral buffered formalin. Formalin-fixed samples were embedded in paraffin wax, sectioned at 5 μm thickness, deparaffinized and stained with H&E dye for 2 min, and quickly dehydrated in 95% absolute alcohol. The H&E-stained tissue sections were observed using an optical microscope Axioimager system equipped for fluorescence illumination.

### 4.6. PTAH Staining

To identify fibrin deposits in lung tissues, small airway specimens were fixed in 10% neutral buffered formalin. Formalin-fixed samples were embedded in paraffin wax and sectioned at 5 μm thickness. The paraffin-embedded tissue sections were deparaffinized and treated with potassium permanganate solution for 5 min and then rinsed thoroughly in distilled water. The sections were bleached in the oxalic acid solution for 2 min, washed in distilled water, and treated with Zenker’s solution containing 5% acetic acid overnight at room temperature. The sections were stained by PTAH solution overnight at room temperature and quickly dehydrated in 95% absolute alcohol. The PTAH-stained tissue sections were observed using an optical microscope Axioimager system for fluorescence illumination.

### 4.7. A549 Cell Culture

Human alveolar basal epithelial cells A549 cells were provided by the American Type Culture Collection (Manassas, VA, USA). A549 cells were cultured in RPMI 1640 supplemented with 10% FBS, 2 mM L-glutamine, 100 U/mL penicillin, and 100 μg/mL streptomycin. A549 cells were sustained in 90–95% confluence at 37 °C in an atmosphere of 5% CO_2_. A549 cells were treated with 1–20 μM astragalin and then stimulated with 10 U/mL thrombin up to 72 h to induce expression of target gene proteins.

Cell viability was determined by using MTT (3-(4,5-dimethylthiazol-2-yl)-2,5-diphenyltetrazolium bromide, Duchefa Biochemie, Haarlem, Netherlands) assay. After removing unconverted MTT, the purple formazan product was dissolved in 0.5 mL 2-propanol with gentle shaking. The absorbance of formazan dye was measured at λ = 570 nm with background subtraction using λ = 690 nm.

### 4.8. DHE Staining for ROS Production

A549 cells (7 × 10^4^ cells) grown on 24-well glass slides were exposed to 10 U/mL thrombin in the absence and presence of 1–20 μM astragalin. A549 cells were fixed with 4% formaldehyde for 10 min and permeated with 0.1% Triton X-100 for 5 min on ice. A549 cells were stained by incubating for 1 h in 20 μM DHE (Invitrogen, Carlsbad, CA, USA). For the identification of nuclei, DAPI was given for 10 min. Stained cells on slides were mounted in a mounting solution. Images of each slide were taken using an optical microscope Axioimager system equipped for fluorescence illumination.

### 4.9. Statistical Analysis

The results were expressed as mean ± SD for each treatment group in each experiment. Statistical analyses were performed using the Statistical Analysis Systems statistical software package (SAS Institute, Cary, NC, USA). Levene’s test was conducted to verify departures from basic assumptions about normality and homogeneity of variance. Significance between groups was determined by one-way ANOVA, followed by Duncan’s multiple range test for post hoc comparisons. Differences were considered significant at *p* < 0.05.

## 5. Conclusions

The current study investigated that astragalin inhibited cigarette smoke-induced pulmonary thromboembolism and alveolar emphysema in mice. Further, this study explored that molecular links between thrombosis and inflammation entailed PAR signaling and oxidative stress-responsive MAPK pathway in thrombin-exposed alveolar cells. The polyphenol astragalin attenuated thrombosis and alveolar damage caused by cigarette smoke, involving modulation of plasminogen activators. Astragalin blocked thrombin-induced PAR signaling leading to alveolar inflammation. In addition, this compound inhibited ROS-mediated activation of MAPK signaling pathways in thrombin-loaded alveolar cells. These data strongly suggest that thrombin can mimic the thrombotic effects of cigarette smoke in evoking thrombosis and inflammation. Furthermore, astragalin as an inhibitor of PAR may reduce the risk of cigarette smoke-associated thrombosis and alveolar rupture. Therefore, astragalin may be a potential agent alleviating alveolar inflammation instigated by pulmonary thromboembolism.

## Data Availability

Not applicable.

## Abbreviations

COPD	chronic obstructive pulmonary disease
COX-2	cyclooxygenase-2
ICAM-1	intracellular adhesion molecule-1
iNOS	inducible nitric oxide synthase
MAPK	mitogen-activated protein kinase
PAR	protease-activated receptors
PAI-1	plasminogen activator inhibitor-1
ROS	reactive oxygen species
TF	tissue factor
tPA	tissue plasminogen activator
uPA	urokinase plasminogen activator
